# Supplementary material for: Are carbon emissions trading and green financial instruments synergistic? -Comprehensive quantitative research based on content analysis
Source: PLoS One. 2024 Mar 7;19(3):e0298601. doi: 10.1371/journal.pone.0298601 (PMC10919601; doi:10.1371/journal.pone.0298601)
Supplement: S1 Data — (ZIP) [file pone.0298601.s001.zip › Data/地级市Z_F工作报告.zip!/║■─╧/╧μ╠╢╩╨/└┤╫╘╣1⁄2╓┌║┼í╛┬φ┐╦╩2╛▌═°í┐_2014.docx]

各位代表:

现在,我代表市人民政府向大会报告工作,请予审查,并请各位政协委员和其他列席人员提出意见。

一、2013年工作回顾

2013年,面对严峻复杂的经济形势,在市委的坚强领导下,在市人大、市政协的监督支持下,我们紧密团结和依靠全市人民,奋发努力,攻坚克难,较好完成了十四届人大一次会议确立的各项目标任务。实现地区生产总值1418亿元(预计数,下同),同比增长11%;财政总收入144.72亿元,增长18.4%;固定资产投资1207亿元,增长35%;社会消费品零售总额398亿元,增长14%;城乡居民收入稳定增长;省定节能减排目标任务全面完成。着力重大战略平台创建,国家级湘潭综合保税区、湘潭国家农业科技园区等获批。湘潭连续五次成为全国科技进步先进城市。湘潭县、韶山市成为全省特色县域经济重点县。基础设施建设、重大产业布局等一系列打基础、利长远的工作取得重要进展。本届政府工作扎实开局。一年来,我们突出抓了以下工作:

(一)突出抓项目建设。着重于强化产业、完善基础、促进转型,全力抓好重点项目建设,项目开工率(含续建)为87%,比上年提高4个百分点,完成投资650亿元,全市固定资产投资连续19个季度保持30%以上增速,投资对经济增长的贡献率达62%。推行重大项目前期工作全程代办,实施全市统一的征拆政策,开展征拆“百日攻坚”,有效保障了项目用地。加大融资力度,全市贷款余额首次突破千亿大关,年新增银行贷款172亿元,发行企业债券36亿元,引进市外投资200亿元,强化了项目要素保障。泰富重工、胜利钢管、塔奥地通等重大项目竣工投产,华拓数码万人级交付基地、昭山晴岚等项目加快推进,万达湘潭广场、威胜智能配用电产业园等一批投资上10亿元项目相继落户,项目支撑发展作用进一步增强。

(二)突出抓实体经济。围绕推进实体经济稳定健康发展,出台稳增长、调结构具体政策,成立国有全资担保公司,开展扶助小微企业专项行动,非公经济发展良好,实体经济企稳向好。规模工业增加值增速扭转下行态势,7月份以来稳定回升,全年增长11%;新增产值过100亿企业1家;规模工业企业利税增长26.9%。服务业发展提速。成功举办先进矿山装备展等会展活动,专业市场、大型城市综合体渐成规模,电子商务快速成长,完成服务业增加值468亿元,增长13%。三次产业结构由上年8.6∶59.7∶31.7调整为7.9∶59.1∶33。园区经济、县域经济快速发展,湘乡市获批国家可持续发展实验区,雨湖区获批国家新型工业化军民结合产业示范基地,岳塘经开区正式起航。

(三)突出抓城市提质。以规划提升带动建设提质。完成湘江风光带修建性详规、昭山风景名胜区总体规划编制;中心城区控制性详细规划实现全覆盖。加快湘江风光带建设,九华段、湘潭县段完成路基工程,高新区段主车道通车;长株潭城际铁路、九华大道、昭山大道等项目顺利推进;五大桥及北引线全面完工,东二环主路基基本贯通,迅达大道、泗洲南路、柏荫路等建成通车,城市框架大幅完善。河西平战结合人防工程主体完工。全面推进“十大环保工程”,竹埠港地区关停重化工企业19家,锰矿区矿山地质环境治理一期、双马垃圾场整改配套工程等顺利竣工。深化“六乱”治理和城区道路交通秩序整治,启动环卫作业市场化。新改建社区公园5个,九华德文化公园基本建成,湖湘公园二期、木鱼湖公园等加快建设,湖湘公园调增面积115亩。推进公交运营体制改革,实现公交回归公益、提质便民。

(四)突出抓“三农”工作。以农业产业化为抓手,推动农业高效发展,完成农业产业化总产值560亿元,增长20%。大力推进土地流转,培育新型经营主体。耕地流转面积达56万亩,流转率居全省前列。农民专业合作社达833家,家庭农场达168家。战胜建国以来最大旱灾,粮食保持稳产,认真执行国家粮食最低收购价政策,全年收购农民余粮42万吨,做到应收尽收。生猪生态养殖、出口外调等稳居全国前列。推进蔬菜基地建设和农超对接,“菜篮子”保障能力进一步提升。深化林权体制改革,林业综合产值达81亿元。推进城乡统筹发展,梅林桥、姜畲两大示范片区建设进展顺利;圆满完成第三轮“百村示范、百村帮扶”工程;韶山核心景区外环公路等4条干线公路建成通车;新建农村公路458公里,完成水利工程3万处,建设高标准渠道420公里,解决21.2万农村人口饮水安全问题。农村环境卫生综合整治全域推进,农村面貌大为改观。城镇化率达到56.52%。

(五)突出抓民生改善。坚持民生优先,全市民生支出占公共财政支出比重提高到62%。省、市为民办实事全面完成。新增城镇就业6.24万人。大学生科技创业园获批国家级创业孵化示范基地。社会保险扩面7万人。启动政府购买养老服务。大幅提高城乡低保补助标准,社会救助水平保持全省领先。基本建成(含竣工)保障性住房和棚户区改造住房11484套,改造农村危房2262户。促进教育内涵发展,合格学校、农村公办幼儿园、校安工程建设等超额完成目标,高中学业水平考试成绩全省第一。医疗服务和保障体系不断完善,新农合住院补偿封顶线提高到12万元,在全省率先实现卫生信息化全覆盖。隆重简朴务实举办纪念毛泽东同志诞辰120周年活动。电视剧《东山学堂》在央视开播。“欢乐潇湘·幸福湘潭”大型群众文艺汇演活动圆满成功。群众体育连续4年保持全国先进。计生工作连续五年保持全省模范行列。开展食品药品安全重点领域专项整治,杜绝了重大安全事故的发生。深化安全生产大检查、大整治,安全生产形势稳定向好。市直机关“群众工作日”活动有效开展,各领域突出民生问题加快化解,社会保持和谐稳定,综合民调居全省第二。

(六)突出抓制度建设。坚持以制度建设规范政务行为。修订完善《市政府工作规则》,健全重大行政决策机制,规范决策程序。建立政府合同审查管理工作联席会议制度,出台政府合同管理办法,加强合同风险防范。制定完善规划管理和实施的五项制度,严格规划调整程序,强化对重点区域和重大项目的规划管理。

实施经营性用地集中统一管理。制定“2+4”征拆政策,征拆市场逐步规范。完善城区财政体制配套措施,切实加强财政预算绩效管理、财政资金审批管理和专项资金管理。完善工程建设领域诚信体系建设,政府投资建设项目工程造价计价等行为得到规范。落实法治政府建设要求,推进政府职能转变,清理规范行政审批和收费行为,推行行政审批集中受理。强化行政监察、审计监督和财政投资评审,加强政府债务清理和风险防范。认真执行市人大及其常委会决议、决定,自觉接受人大、政协和社会各界监督,办理人大代表建议和政协委员提案762件,办结率100%。妇女儿童、老龄、机构编制、民族宗教、工商、质监、广电、档案、移民、气象、水文、韶灌、石化、石油等工作取得新进展。

各位代表,成绩来之不易。这是在中共湘潭市委的正确领导下,全市上下深入贯彻落实中央和省委省政府重大决策部署、奋力拼搏的结果,是社会各界关心支持的结果。在此,我代表市人民政府,向辛勤工作在各行各业的广大干部群众,向人大代表、政协委员,向各民主党派、工商联、人民团体,向无党派人士和离退休老同志,向驻潭人民解放军、武警官兵和政法干警,向所有关心、支持湘潭改革发展的各界人士,表示衷心的感谢!

在总结成绩的同时,我们更看到一些问题仍然比较突出:发展不充分是目前最大的问题,经济总体实力偏弱,结构不优,增长和转型的基础不牢。社会就业和增收渠道不宽,居民收入水平提高不快。城市经营水平和投资效益不高,规划执行不严,建设方式比较粗放,老城区、老工业区提质改造任务繁重,交通堵塞、停车难等现象突出,城市整体功能不强。公共服务与社会保障水平仍不够高,群众在就学、就医、出行、住房等方面仍存在较大困难。农村基础设施建设仍相对薄弱,农业综合生产能力和抗风险能力不强。环境保护薄弱环节多,部分领域和地段环境问题仍不时出现。一些干部大局意识、服务意识、担当精神不强,行政审批和收费还存在较为严重的问题,发展环境还需大力优化,等等。对此,我们必须拿出具体措施,切实加以解决。

二、2014年工作目标、任务和重点

2014年是全面深化改革、加快转型发展的重要一年。我国经济已进入以质量、效益为中心的中高速发展阶段。我市经济企稳回升,尽管基础还不太牢固,但总体呈平稳增长态势。我们将全面贯彻落实党的十八大和十八届二中、三中全会精神,按照中央、省委、市委经济工作会议的要求,以科学发展、富民强市为主题,以加快转变经济发展方式为主线,以提前全面建成小康社会为目标,坚持稳中求进、改革创新,深入实施“四化两型”战略,加快推进“两个率先”、“三量齐升”,着力深化重点领域改革,着力推进产业转型升级,着力完善基础设施建设,着力提高群众生活水平,努力建设幸福湘潭。

主要预期目标是:地区生产总值增长10.5%;固定资产投资增长25%;规模工业增加值增长12%;社会消费品零售总额增长13.5%;进出口总额增长14%;财政总收入增长12.5%;城乡居民人均收入分别增长11%和12%;万元GDP能耗下降3.66%,主要污染物排放总量削减完成省定目标。突出抓好十项改革任务、十大产业项目、十大基础工程、十大民生实事。

十项改革任务:深化行政体制改革和事业单位分类改革;深化财税管理体制改革;深化企业改革改制;深化医药卫生体制改革;深化公交体制改革;推进投融资体制改革和金融改革;推进土地管理制度改革;推进文化体制机制创新;推进教育领域综合改革;健全城乡发展一体化体制机制。

十大产业项目:国家级湘潭综合保税区;吉利汽车零部件产业园和上海大众汽车配件产业园一期;威胜智能配用电产业园;湘潭电化集团新产业园;中国(中部)国际商贸城;金阳农产品商贸物流城;泰富重工二期;万达湘潭广场;义乌小商品城;华拓数码万人级交付基地。

十大基础工程:湘江风光带;竹埠港地区重金属污染治理和锰矿地区环境整治;二环线、潭锰路等城市干道工程;G320城际快车道(含昭华大桥)、昭山大道等国省干线、农村公路及危桥改造工程;市民之家(市政务中心);城区标识标牌、派出所建设;爱劳渠综合整治工程;长株潭城际铁路及配套工程;沪昆高铁湘潭北站、韶山南站工程;雨湖公园提质改造、绿道建设工程。

十大民生实事:新增城镇就业5万人,政府出资购买公益性岗位和运用岗位补贴解决困难人员就业2000名,新增农村劳动力转移就业1.5万人;新建廉租住房和公共租赁住房各1000套,农村危房改造1000户,资助农村特困户建房200户;免费为贫困残疾人适配助听器300例;对城乡60岁以上失独老人实施扶助,改扩建农村敬老院8所;继续实施百村帮扶工程,建设农村生活垃圾整治示范村20个,新解决农村饮水不安全人数10万人;提质改造市城区5个老社区基础设施;新建农村义务教育合格学校65所、农村公办幼儿园9所;完善乡镇广场文化、体育健身设施30处,扶持建设城乡社区文化活动室30个;推进肉菜流通追溯体系建设,新扩无公害蔬菜专业基地1000亩;新安装治安视频监控摄像机800个。围绕上述目标任务,全力抓好以下五方面工作:

(一)大力推进改革创新,加快转变发展方式。把改革创新作为今年的当头大事,充分挖掘“两型社会”试验区先行先试政策潜力,务求实质性突破,促进发展加快转型。

1、积极稳妥推进改革。严格按照中央要求,适时适度,分类推进。对目标明确、可以操作的改革,加快实施。投融资方面,规范政府投资行为,建立投资项目后评价、重点项目公示和责任追究制度;完善政府投融资公司运营机制,提高自主经营能力。金融信贷方面,加快发展多层次资本市场体系,强化金融对实体经济的支持;鼓励和扩大民间资本进入金融服务领域,规范发展小额贷款公司、担保公司等;完善金融信贷风险防范机制,规范民间借贷行为,严厉打击金融违法犯罪活动,优化金融生态环境。财税管理方面,进一步规范、微调城区、开发区、示范区的分配体制,适当提高市本级的宏观调控能力;推进现代预算管理、财政性资金绩效评价、政府性债务管理、财政支出结构调整等改革;深化“营改增”和房产税试点。企业改革方面,全面启动104户企业改革改制,本届政府任期内全面完成;推进企业产权改革,大力发展混合所有制经济;完善现代企业制度,建立职业经理人制度,健全法人治理结构;完善国有企业激励约束和经营投资责任追究机制等。对必须推进、试验示范的改革,试点先行。探索建立城乡统一的建设用地市场,推动土地“二次开发”;完善节约集约用地机制,实行招商引资项目用地指标与投资、纳税强度相挂钩,探索工业用地租赁模式;依法全面规范经营性用地管理;妥善处理征拆安置等历史遗留问题。开展市政基础设施领域引进社会资本试点。探索城乡校长、教师流动轮岗机制和联合办学、对口支援办学机制;开展“职业技能振兴”试点,推进国家级创业孵化示范基地建设,探索大学生创业就业新机制。完善农业补贴和保险制度,开展蔬菜、生猪价格政策性保险试点和农村小额信用贷款试点,建立政府主导的农业信用担保体系。对完善制度、巩固深化的改革,深入推进。巩固完善基本药物制度,重点抓好基层卫生室实施基本药物制度;深化基层医疗卫生机构综合改革,健全稳定长效的多渠道补偿机制;推进公立医院综合改革,构建医联体服务新模式。推进户籍管理改革,合理确定市城区落户条件,有序放开落户限制;稳步推进农业转移人口市民化,优先解决存量,有序引导增量,重点解决已转移到城镇就业的农业转移人口落户问题。完善土地承包经营等权利的抵押、担保权能;建立健全农村土地流转服务体系。有序推进廉租房、公共租赁房并轨,完善保障性住房管理制度。完善文化管理体制;建立健全现代文化市场体系,推进国有经营性文化单位转企改制,建立多层次文化产品和要素市场;完善文化生产经营机制,加快发展文化产业。

2、突出创新驱动发展。加强重大产业关键技术攻关,重点突破先进装备、新能源、汽车关键零部件、新型特种钢等核心关键技术。强化科技成果转化,促成产学研合作项目40项以上。加快建设“智慧湘潭”综合平台,提高信息化应用水平。推动信息化和工业化深度融合,打造10家两化融合示范企业和50家数字企业。强化企业在技术创新中的主体地位,支持企业组建各类创新平台,实施一批重大科研项目,推进先进矿山装备、风电装备、汽车及零部件、湘莲等产业技术创新战略联盟建设,抓好军转民技术和产品应用。深化校地合作、校企合作、产教融合,促进大学科技园创新发展,支持高校院所科技成果入园转化,支持高校面向产业和区域协同创新。加强创新型人才队伍建设。推进“质量强市战略”。加强知识产权运用和保护,提高知识产权转化率。

3、强化消费拉动增长。加快市场体系建设,提升城区商业网点规划,优化高端消费平台布局。充分发挥步步高、心连心等大型连锁企业带动作用,推进河西中央商务区、建设路口、板塘铺、砂子岭等商圈建设。继续实施农贸市场和农产品直销店建设三年行动计划,深入推进“万村千乡”市场工程和县乡流通再造工程。年内完成15个农贸市场改造和标准化建设、120个农产品直销店建设。稳定汽车、住房等大宗消费,大力培育健康、文化、信息等消费新增长点,促进消费升级。有效组织房交会、车博会等会展活动,打造消费热点。加强消费市场监管,深入开展“诚信兴商”示范创建,维护市场稳定和消费安全。

4、提高开放发展水平。推进湘潭综合保税区一期建设,创新体制机制,确保年内封关运行。加快韶山出入境检验检疫局建设。巩固传统出口市场,稳定骨干企业进出口增量,抓好华菱湘钢等12家企业外贸“一对一”帮扶,推动吉利汽车自营整车等优势产品出口。大力开拓东盟、南非、南美等新兴市场,扶持新型出口“拳头产品”,支持有条件的企业“走出去”。推动外贸转型升级,依托国家外贸转型升级示范基地和科技兴贸基地,培育壮大外贸主体,打造一批具有自主知识产权和品牌的出口产品。大力发展服务贸易,年内培育和引进服务外包企业20家以上。

5、坚持绿色发展模式。推进清洁生产,大力推广十大清洁低碳技术。年内淘汰水泥、造纸、铁合金等落后产能15万吨,关停“五小”企业20家。落实《湖南省湘江保护条例》实施方案,以湘江保护“一号重点工程”为龙头,以“保证水量、优化水质、改善生态、畅通航道”为总目标,深入推进“十大环保工程”。实施湘江长沙综合枢纽湘潭库区移民安置部分专项工程。抓好河道采砂整治、危险废物专项整治、养殖业污染治理等工作。湘江湘潭段全面禁止采砂,沿岸禁养区养殖场3年内全部清退。城市污水处理率达到88%,水功能区水质达标率达到70%以上,让人民群众喝上干净水、放心水。加强工业烟气、车船排放污染和扬尘治理,空气质量优良率提高5个百分点。扩大排污权交易试点范围,建立排污权有偿使用机制。推动建立昭山生态补偿制度。推进餐厨废弃物资源化利用和无害化处理国家试点城市建设。推广可再生能源建筑应用120万平方米,完成建筑节能改造8万平方米。城市公交普及新能源车,建设公共自行车租赁系统,构建“绿色出行”体系。推进“绿色湘潭”建设,创建国家生态园林城市。抓好新造林、封山育林和森林抚育,全市森林覆盖率达到46.1%。加强湘江湿地、水府庙湿地和生态环境保护。

(二)牢牢抓住经济建设中心,全面增强综合竞争实力。坚持经济建设中心地位不动摇,着力提高发展的质量和效益,全面增强区域综合竞争实力。

1、抓好重点项目建设,夯实经济发展后劲。年内安排重点项目240个左右,完成投资780亿元以上,全市完成固定资产投资1500亿元。突出抓好重大产业项目。力争红星美凯龙、华侨城、广州富力、湘潭雅礼中学、宋庆龄基金会幼儿园、一力物流等项目签约落地,全创科技二期、福星国际金融中心等项目竣工。健全重大项目前期开发工作机制,市本级安排重大前期项目100个,确保有15个以上开工建设。完善项目协调推进机制,严厉打击阻碍项目建设的违法犯罪行为。强化项目要素保障。引进市外投资220亿元以上,新增银行贷款200亿元以上。支持企业上市融资。大力促进民间投资。积极争取用地指标和补充耕地指标;强化建设用地监管,清理处置闲置土地,优化用地结构,科学设置开发强度,提高土地利用效率;全面落实征拆政策,提高供地率,确保重点项目用地需求。

2、着力提升产业素质,增强实体经济实力。促进工业链群发展。坚持新型工业化第一推动力不动摇,着力完善产业链,推进产业集群。支持企业围绕完善产业链布局项目;积极开展产业链招商,重点引进高端装备项目、汽车零部件配套企业、电子信息整机企业、风电产业配套项目、节能环保产业项目等;抓好湘潭电化新产业园等项目建设。推动企业做大做强,重点培育50家成长型工业企业,力争新增产值过亿元企业20家,过10亿元企业5家,过50亿元企业1家以上。优化国有企业结构布局,支持国有企业转型发展,带动一批产业链关联企业发展壮大。鼓励、引导、支持非公经济加快发展,实施中小企业“百千万”成长工程,完善中小微企业服务平台,支持企业开拓市场,加快打造一批“小巨人”企业。促进服务业规模发展。推进服务业发展提速、比重提高、水平提升。加快商贸集聚区、物流园区和专业市场建设,重点抓好岳塘经开区商贸物流集聚区、湘潭金融商务区等项目建设。加快发展电子商务、会展经济、楼宇经济等新型业态,推进湘潭中心大厦、中国网库等项目建设,积极引进品牌电商,举办10个以上重点会展活动。推进韶山旅游综合服务中心、水府旅游区、窑湾历史文化街区、昭山风景区提质改造等项目建设,加快创建全国红色旅游综合发展示范区。促进农业创新发展。加快构建新型农业经营体系,推进农业发展方式转变,以高产、优质、高效、生态、安全为目标,大力发展都市农业。重点实施粮食产业提升等“现代农业十大工程”。坚持数量、质量并重,稳定粮食种植面积,提高粮食综合生产能力;推进市、县粮食收储体系建设和“危仓老库”提质改造,支持粮食龙头企业发展精深加工,提高粮食收储能力;促进规模经营,农村耕地流转70万亩,农民合作组织发展到1000家,家庭农场发展到200家以上;大力发展农产品加工业和品牌创建,促进增效增收,农业产业化总产值突破630亿元。开展“农业项目建设和招商年”活动,策划包装100个农业重大项目,促进一批项目落地实施。以湘乡市为主体核心区,加快湘潭国家农业科技园区建设。

3、统筹区域经济发展,营造多极支撑格局。推动园区转型发展。深化园区发展机制创新,强化产业支撑,节约集约使用土地等各种资源要素,园区规模工业增加值增长15%以上。积极探索“一区多园”发展模式,鼓励跨区域共建,拓展发展空间。加强园区公共服务平台建设,完善生活配套设施,增强园区承载能力。加强园区产业布局引导,避免同质竞争。高新区、经开区重点突出新型工业发展,不断壮大新能源、先进装备制造、汽车及零部件、电子信息等产业。昭山示范区重点发展健康养生、文化创意、高端商务、生态旅游等新兴服务业,加快绿色崛起步伐。推动老工业区提质发展。高度重视老工业区发展,重振老工业区发展活力。制定老工业区重振方案。积极对接全国老工业基地调整改造政策,争取国省资金和项目。大力改善老工业区环境,抓好重型车辆行驶专用道路等项目建设,完善基础配套设施。竹埠港化工区全面完成化工企业关停任务,加快实施整体退出和环境综合治理。锰矿地区重点推进矿山地质环境治理示范工程二期、国家矿山公园、废渣废水治理、供排水设施、白云路等项目建设,加快湘潭电化整体搬迁。下摄司循环产业基地重点加快钢铁工业节能减排和产品结构调整。湘乡重化工区重点推进五矿湖铁、湘乡氟化学等企业转型提升,打造全省循环经济试点示范区。推动县域经济加快发展。坚持园区经济、县城经济、乡镇经济“三位一体”,壮大县域经济总体实力。着重发展县域园区,支持园区依托特色定位,引进和建设一批重大产业项目,支持县域园区与湘潭高新区、经开区配套协作。推行“园区加生产基地”模式,带动区域主宗农产品加工升值。大力发展特色产业,推进湘潭县、韶山市两个全省特色县域经济重点县建设。推动县域中心城市扩容提质,带动三产业加快发展。大力发展乡镇经济,引导乡镇集中力量抓项目、抓产业,支持县市区采取“一区多园”方式发展乡镇工业,增强乡镇造血功能,努力建设一批经济强镇。

(三)突出城市基础设施建设,打造宜居宜业家园。深入实施“北进中强”方略,加强城市基础设施配套和功能完善,加大城市经营力度,提高城市品质,让居民生活更加舒适。

1、注重规划效益,加强规划统领。强化规划的科学性、权威性和严肃性,实行集中统一、最严格的规划管理。城市规划逐步由扩张性规划转向限定城市边界、优化空间结构的规划。启动新一轮城市总体规划修改工作,发挥总体规划的统筹引领作用。开展特色湘潭规划,编制完成城市色彩、景观风貌等规划;抓好湘江两岸城市设计和城市主入口空间设计,扮靓城市空间。推进宜居湘潭规划,编制完成城市排水防涝等规划和杨梅洲、宝塔公园规划,优化河西交通网络与城市基础设施配置,调整优化昭山片区总体规划。构建公众了解、参与、监督规划的平台,规划展示馆开馆,规划一站式服务和三维辅助审批系统投入使用;创新和完善动态化、网格化的规划管理机制,保持规划连续性,严格查处和整治违法违规建设,确保城市建设按规划实施。

2、注重设施配套,加强城市建设。安排城市建设重点项目63个,年度完成投资252亿元。加大经营城市力度,抓好公共资源、基础设施项目及无形资产等的经营开发。加快推进湘江风光带建设,九华段、高新区段年内通车。加快打开对外通道,推进沪昆高铁、长株潭城际铁路和益娄高速、长株潭外环高速、长韶娄高速建设。加快城际道路建设,九华大道、昭山大道、昭华大道建成通车,启动G320城际快车道等项目建设。启动河西中心港综合水上服务中心、湘潭市旅游客运中心港建设,力争铁牛埠港区二期建成运行。加快完善城区路网,力争羊牯大道、东二环、书院东路等道路竣工通车,启动月华北路、一环东路、宝塔南路等道路建设。抓好一大桥、三大桥引线改造,拉通大湖南路。加快站场和停车位建设,推进湘潭新汽车西站、湘潭客运中心站、韶山综合客运枢纽中心站的建设,启动马家河物流中心和大型商场周边停车位建设。推进城区机关事业单位非经营性停车场、厕所免费对社会开放。加大老城区改造力度,推进“城中村”和棚户区改造,全面提质老旧社区、老旧厂区环境。提质改造砂子岭、富洲路等城市出入口。完成东二环、迅达大道等城市道路绿化,植树补绿向次干道、小街巷、居民区延伸。提质改造排水防涝设施,实施下摄司片区、万楼片区、河西旧城区排水改造等项目。完善污水处理设施,完成九华污水处理厂、河东河西污水处理厂提标改造、湘江排污口截流等项目建设,启动河东第二污水处理厂及配套截污管网建设。加快垃圾处理场所建设。完成爱劳渠综合整治、木鱼湖、和平公园二期以及5个以上社区公园建设,启动宝塔公园、仰天湖公园建设,抓好二大桥桥头绿化提质改造。整治提升雨湖公园,取缔公园内商业性经营活动,使公园真正成为老百姓活动的场所。3、注重群众关切,提升管理水平。从群众最关心的问题抓起,坚持重点治理与日常管理相结合,大街小巷与住宅区相结合,城市中心区与城乡结合部相结合,横向到边,纵向到底,推进城市管理全覆盖、长效化,创建全国文明城市和卫生城市。加强城市交通源头治理,解决重点交通堵点问题,保障交通畅通。划定定点经营区域,对夜市和流动摊贩实行规范管理。开展建筑工地及渣土运输、混凝土搅拌站场及运输、油烟污染、噪音污染、“车窗抛物”等专项整治,完成数字城管系统建设,全面推广环卫服务市场化,提升城市管理网格化、精细化和标准化水平。深化出租车行业文明服务创建,大力整治出租车拒载、强行拼车、乱喊价等行为。严厉整治非法营运。突出公共交通的公益属性,巩固公交资源整合成果,建立财政补贴补偿机制,实施老年人、残疾人、军人免费乘车和学生、市民刷卡乘车票价优惠。调整公交线路,完善站场配置,优化运营服务,提高公交覆盖率和市民出行公交分担率。湘潭县城与市区实现公交同城。启动城乡客运一体化建设,逐步开通市城区至县城及中心镇的公共交通。

(四)推进城乡统筹发展,努力建设“美丽乡村”。按照城乡发展一体化要求,统筹推进各项建设,营造城镇相连、交通便捷、经济发展、生态宜居的城乡和谐空间。

1、加快城乡基础设施对接。推进国省干线公路建设,干线公路与农村公路有效对接,湘乡至壶天一期、潭锰路等干线年内建成通车。新建农村公路286公里、农村客运招呼站50个,改造危桥34座;加强农村公路管理和养护,实施国省干线公路和农村公路的大中修、公路路网结构改造工程、文明示范路创建等,加强车辆超限超载治理,提高公路安全通行能力。加快农村信息化建设,推进农村“三网融合”。推动市、县城区供水、燃气网络向周边农村延伸。加强农村电网扩容改造。加强农村“五小”水利工程、中小型水库除险加固和城市堤防建设,年度完成投资12亿元。推进土地综合整治,加强高标准农田建设和中低产田改造,提高耕地综合生产能力。

2、推进特色镇建设。启动3个特色镇建设。坚持以人为核心和尊重自然、顺应自然、天人合一的理念,保护现有山水脉络等独特风光,保护和弘扬传统优秀文化。年内编制完成镇域一体化规划、总体规划、控制性详细规划和产业规划。先行畅通镇区内外交通,加快建设特色镇连接市区、县城、国省道及高速公路的快速交通干线,改善特色镇通乡通村路网结构。加快特色镇自来水厂、污水处理厂、垃圾中转站、综合市场、主干道、中心广场等基础设施建设。积极培育特色优势产业,策划、包装一批重点产业项目,积极开展招商引资,力争部分项目落地。

3、建设“美丽乡村”。深化农村环境卫生整治,全面完成“三年整治行动”任务。推进梅林桥“美丽乡村”示范片、姜畲现代农业示范区和新农村示范片建设,重点抓好梅林大道、姜畲观光大道提质工程、紫荆河二期等项目建设。推动水利建设与观光旅游相结合,启动韶山银河旅游区建设。推进扶贫开发,加快边远山区、库区基础设施建设,加大移民脱贫致富帮扶力度。大力实施农村清洁工程,抓好户用沼气池等农村清洁能源建设。在改造农村环境中,注重保留村落原始风貌,慎砍树、不填水、少拆房,尽可能在原有村落形态上改善居民生活条件,打造生态自然、生产高效、生活美好、人文和谐的“美丽乡村”。

(五)突出保障和改善民生,促进社会和谐发展。按照“守住底线、突出重点、完善制度、引导舆论”的要求,推进民生社会建设。

1、健全民生保障体系。坚持以创业带动就业,深入推进“国家级创业型城市”建设,完善扶持创业各项政策,全年新增创业3000人,开展职业技能培训10万人次以上,重点抓好高校毕业生就业和产能调整、企业改革改制产生的下岗人员再就业,推动实现更充分、更高质量的就业,确保城镇登记失业率控制在4.3%以内,零就业家庭动态就业援助达100%。整合城乡居民基本社保体系,实施5万人扩面工程,社保、医保全面提标,推进社会保障由制度全覆盖向人群全覆盖转变。提高城乡低保、农村五保救助标准,加强城乡低保资金监管。加强保障性住房和安置房建设,新增各类保障性住房和棚户区改造住房1.5万套(户)。落实惠民政策,多渠道增加居民财产性收入,逐步提高最低工资标准,完善工资支付保障制度,促进城乡居民持续增收。

2、提升基本公共服务水平。优化城乡义务教育资源均衡配置,提高农村教师待遇,大力促进教育公平;推进教育信息化,建成教育管理公共服务平台;实施“校园安全”工程;启动全国示范性综合实践基地建设。完成九华和平小学、昭山和平小学新建及许家铺学校、火炬小学的扩建提质任务。繁荣公共文化,提升“欢乐潇湘·幸福湘潭”等群众文化活动品牌,完成市博物馆布展并对外开放,完善公共文化服务体系,不断满足人民群众文化需求。统筹城乡卫生一体化发展,抓好市中心医院儿童诊疗中心和全科医生临床培养基地、基层卫生室等项目建设,加强城乡居民健康教育,推进中医药事业和健康服务业发展,提高城乡居民重大疾病保障水平,扎实做好疾病防控、新生儿出生缺陷干预等工作。积极发展体育事业,完成体校重建并投入使用。对中心城区道路名称进行确认或命名,逐步设置新型路名牌,启动门牌号编码和设置工作。加快构建社会化养老服务体系,发展养老产业。健全农村留守儿童、妇女、老年人关爱服务体系,大力发展残疾人、福利和慈善事业。坚持计划生育基本国策,启动一方是独生子女的夫妇可生育两孩政策。坚持计划生育党政领导负总责和“一票否决”,稳定低生育水平,提高出生人口素质,促进人口长期均衡发展。进一步抓好民族宗教、外事侨务港澳、台务、供销、地震、气象、保密等工作。加强国防动员、民兵预备役和人防工作,推动军民融合深度发展。深入开展全民国防教育和“双拥”共建活动,巩固发展军政军民团结局面。

3、全面加强社会治理。抓好第九届村委会换届选举,加大对农村基层的投入力度,逐步提高村干部待遇。全面启动社区警务室建设,完成市城区16个派出所和10个无房社区办公用房建设,夯实基层基础。加强社会组织培育管理,鼓励民间公益组织发展。完善突发事件应急管理机制。健全防灾减灾救灾体制。加强和完善信息网络管理。建立健全社会征信体系。理顺监管职能,加强基层执法力量,完善统一权威的食品药品安全监管机构,建立最严格的覆盖全过程的监管制度,保障食品药品安全。坚持安全生产高标准、严要求,全面落实安全生产责任制和责任追究制,深化安全生产大检查,强化隐患排查治理和“打非治违”,做到“全覆盖、零容忍、严执法、重实效”,坚决遏制重特大安全事故。建立畅通有序的诉求表达、心理干预、矛盾调处、权益保障机制,推行网上受理信访制度,着力化解社会矛盾。深入推进法治湘潭建设,加强司法救助和法律援助。抓好特殊人群收治、管理。加强社会治安综合治理,推进治安防控社会化网络化信息化建设,深入开展专项打击和重点整治,依法严密防范和惩治各类违法犯罪活动,维护社会和谐稳定。

三、持续推进服务型政府建设

按照“职能科学、结构优化、廉洁高效、人民满意”的要求,加强法治政府和服务型政府建设,切实提高政府服务发展、服务民生的能力。

坚持以发展为大,加快转变政府职能。按照中央“使市场在资源配置中起决定性作用和更好发挥政府作用”的要求,大力简政放权,该放的彻底放开,该管的切实管好。推进行政审批制度改革,进一步取消、下放行政审批事项,市场、社会和基层能办好的事情坚决放开。严禁一切违规审批和收费,行政事业性收费按下限固化收取,规范与行政审批关联的中介机构、社会组织行为,依法取消这类机构和组织依附行政管理的收费。政府将主要精力集中到加强发展战略、规划、政策、标准的制定实施和市场监管、公共服务、社会管理、环境保护上来,营造稳定、透明、公平的发展环境。启动政府机构改革,优化政府组织结构和职能配置,完善行政运行机制。推进事业单位分类改革,加大政府购买公共服务力度,推动公办事业单位与主管部门理顺关系和去行政化,推进有条件的事业单位转为企业或社会组织。严格控制机构编制,财政供养人员只减不增。加强公务员培训,提高适应新形势的能力。坚持正确的政绩观,大力改进工作作风,严格绩效管理,突出责任落实,强化政府公信力和执行力。

坚持靠创业图强,热忱为企业搞好服务。富民强市是湘潭之梦,创业是富民强市之基。依靠企业全力创业,聚集起强大的物质力量,是湘潭富强之路的关键所在。我们要把服务企业发展作为强市之路、富民之举,以足够的热情和精力倾注于企业。注重结合企业实际开展政策研究,加大政策落实力度。依法依规加强对企业的帮扶,努力帮助企业破解生产经营难题。实行统一的市场监管,清理和整治各类违法实行优惠政策行为,建设法治化营商环境。积极搭建银企对接、对外开放交流等各类平台,支持和帮助企业拓展发展空间。依法保护企业家合法权益。加快市政务中心建设,完善各级政务服务体系,优化服务流程,拓宽集中审批、并联审批范围,推行审批项目“两集中、两到位”,提高办事效率。严格整治各种涉企收费行为,推行基本建设项目“一表制”收费,切实减轻企业负担。严格执行涉企检查报备、企业“宁静日”、同城一级检查等制度,加强对重点项目、企业周边环境的专项整治,让企业免遭干扰、舒心发展。

坚持走群众路线,真心实意为百姓着想。深入开展党的群众路线教育实践活动,把自己的命运和群众的命运紧紧连在一起,同呼吸,共甘苦。多站在老百姓的角度看问题、想事情,从上学难、看病难、就业难、出行难等这些老百姓深感烦心的事情抓起,政府多一分努力,百姓就少一分困难。在对待群众的问题上,我们不搞夸夸其谈,说一百句漂亮话不如干一件实事。因此,尽管目前经济还较困难,我们也一定要设法压缩其他开支,集中更多财力用于加大民生支出、帮扶困难群众。坚决做群众利益的代言人和保护者,文明执法,公正办事,及时纠正违法或不当行政行为,打造诚信政府,促进社会公平正义。深入开展纠风和专项治理,重点纠正金融、电信等公共服务行业和教育、医疗、涉农、征地拆迁、涉法涉诉等领域损害群众利益的不正之风和突出问题。健全群众诉求表达机制,发挥“12345”市长热线等平台功能,诚恳接受群众对政府工作的监督和评议。加快基层便民服务中心规范化建设,努力使城乡居民就近享受公共服务。

坚持用制度管人,切实加强廉政建设。全面落实廉洁从政各项规定,推行政府及其工作部门权力清单制度,依法公开权力运行流程,坚持用制度管权管事管人,从源头上预防和治理腐败。健全权力监控、行政过错追究和纠风长效机制,坚决反对滥用职权、以权谋私。依法管理经济社会事务,落实议事规程和决策程序,自觉接受人大、政协和社会各界监督。重大决策出台前向人大报告。推进行政协商,重要决策听取政协意见。厉行节约,反对浪费,坚持从严从简、勤俭办一切事业,严格执行《党政机关厉行节约反对浪费条例》、《党政机关国内公务接待管理规定》,“三公”经费只减不增。深入推进重点领域政府信息公开,财政预决算和“三公经费”严格按规定公开。加强行政监察和审计监督,严肃查处违法违纪人员。坚持廉洁从政从我做起。在此,我与政府全体工作人员,郑重约法三章:坚决不允许在项目建设、工程承包、招标投标等工作中搞暗箱操作;坚决不允许任何人滥用自由裁量权,损害执法公正;坚决不允许任何人违规收受或赠送礼金礼品,损害廉洁从政。政府领导班子成员率先遵守、以身示范。请各位代表、政协委员和社会各界严格监督。各位代表!时代赋予重任,实干成就梦想。让我们在中共湘潭市委的坚强领导下,凝聚起全市人民的智慧和力量,奋勇争先,艰苦创业,为实现率先统筹城乡发展、提前全面建成小康社会、加快幸福湘潭进程而努力奋斗!
